# Sodium propionate modulates gut microbiota and blood parameters in healthy mice

**DOI:** 10.3389/fmicb.2025.1670591

**Published:** 2026-01-14

**Authors:** Wenjie Cheng, Junhong Zhu, Lanlan Yi, Guangyao Song, Yuxiao Xie, Shuailiang Che, Sumei Zhao

**Affiliations:** Faculty of Animal Science and Technology, Yunnan Agricultural University, Kunming, China

**Keywords:** sodium propionate, gut microbiota, histopathology, blood parameters, healthy mice

## Abstract

**Introduction:**

Short-chain fatty acids (SCFAs), particularly propionate, play crucial roles in host metabolism, immunity, and gut barrier function. However, the direct physiological effects of propionate on healthy organisms remain unclear. This study investigated the impact of sodium propionate (SP) supplementation on intestinal barrier function, gut microbiota, and hematological parameters in healthy C57BL/6 mice.

**Methods:**

Mice were orally administered 200 mg/kg SP for 21 days.

**Results:**

Results showed no significant changes in body weight, intestinal histopathology, or blood parameters. The immunohistochemical results showed decreased IL-6 expression, while IL-10 and occludin expression were increased. Gut microbiota analysis revealed decreased α-diversity in the SP group, along with shifts in microbial composition, including increased *Akkermansia* and *Bacteroides*. No significant differences in colonic SCFA concentrations were observed.

**Conclusion:**

These findings suggest that propionate modulates gut microbiota and hematological parameters in healthy mice, providing insights into its physiological roles under normal conditions.

## 1 Introduction

Short-chain fatty acids (SCFAs) are key components of gut microbiota-derived metabolites and have become an area of intense research interest due to their physiological functions (Morrison and Preston, 2016; Nireeksha et al., 2025). SCFAs are produced by gut microbial fermentation of dietary fiber and primarily consist of acetate, propionate, and butyrate. These SCFAs exert beneficial effects on host health through various mechanisms (Mukhopadhya and Louis, 2025). They play critical roles in inhibiting oxidative stress, suppressing pro-inflammatory cytokine expression, modulating innate immune cells, and enhancing intestinal barrier function (Tong et al., 2016; Mann et al., 2024). SCFAs exert their effects via receptors such as GPR43, GPR41, and GPR109A, and also regulate physiological processes by inhibiting histone deacetylases (HDACs), thereby promoting histone acetylation (Zietek et al., 2021). Among SCFAs, butyrate has been the most extensively studied, whereas research on propionate remains relatively limited.

Propionate is one of the key SCFAs and plays unique roles in metabolism, immunity, and disease regulation. Studies demonstrate that propionate modulates energy metabolism by activating the APN-AMPK-PPARα signaling pathway, thereby exerting therapeutic effects against fatty liver disease (Yang et al., 2023). Propionate has been demonstrated to enhance insulin sensitivity (Liu et al., 2025). Reduced propionate levels are closely associated with glucocorticoid overuse-induced disorders of glucose and lipid metabolism (Zhang et al., 2024). Additionally, propionate may exhibit potential anti-tumor effects. Research has demonstrated that propionate induces accumulation of reactive oxygen species (ROS) in cervical cancer cells, suppresses the NF-κB and AKT/mTOR signaling pathways, ultimately triggering autophagy and inhibiting cancer cell survival (Pham et al., 2021). Propionate can also suppress influenza-induced pulmonary inflammation by increasing Foxp3^+^ cells and IL-10+ cells in splenic CD4^+^ T cells (Kato et al., 2023). Furthermore, propionate influence neurodegenerative diseases and has therapeutic effects on cardiovascular diseases. It improves vascular calcification by modulating gut microbiota such as *Akkermansia* via the gut-vascular axis (Yan et al., 2022). Propionate also regulates helper T cell homeostasis, thereby reducing cardiac hypertrophy, fibrosis, susceptibility to arrhythmias, and atherosclerotic lesion burden. Furthermore, it significantly alleviates systemic inflammation, decreasing the frequency of effector memory T cells in the spleen and the population of Th17 cells (Bartolomaeus et al., 2019).

The intestine serves as the primary organ for nutrient digestion and absorption and is also recognized as the body’s largest immune organ (Horn and Sonnenberg, 2024; Bai et al., 2025). The intestinal barrier provides robust protection against harmful internal and external microorganisms, and its dysfunction is associated with various diseases, including non-alcoholic fatty liver disease (NAFLD), type 2 diabetes (T2D), and inflammatory bowel disease (Neurath et al., 2025; Pena-Duran et al., 2025). Propionate ameliorates DSS-induced colitis in mice by reducing inflammation and oxidative stress via the STAT3 signaling pathway, thereby improving intestinal barrier function (Tong et al., 2016). Another study demonstrated that propionate enhances intestinal barrier integrity through the AKT pathway and improves motor behavior in Parkinson’s disease model mice (Huang et al., 2021). Mechanistically, propionate binds to G protein-coupled receptor 43 (GPR43) on intestinal epithelial cells and increases histone acetylation, upregulating the expression of tight junction proteins (occludin and ZO-1) and boosting mucin production. These effects collectively strengthen epithelial barrier integrity and mitigate radiation-induced intestinal injury (He et al., 2023). However, the multifunctionality of propionate and its underlying mechanisms warrant further investigation.

However, the direct physiological effects of propionate on healthy organisms remain incompletely understood. Therefore, elucidating the impact of propionate on normal physiological systems under disease-free conditions holds significant theoretical value, as it may provide foundational evidence for subsequent disease intervention studies. In this study, we selected healthy C57BL/6J mice as a model and administered sodium propionate via oral gavage to investigate its effects on the intestinal barrier in normal physiology. This work represents the first systematic evaluation of propionate’s comprehensive effects under normal physiological conditions, providing novel perspectives for understanding the physiological functions of SCFAs.

## 2 Materials and methods

### 2.1 Animal experiments and sample collection

A total of 12 male C57BL/6 mice (weight: 19–21 g) were purchased from the Laboratory Animal Center of Yunnan University. All mice had free access to water and food, were housed at 22 °C ± 2 °C under a 12-h light-dark cycle. The mice were acclimatized for 7 days and then randomly divided into two groups: a control group and a sodium propionate (SP) group (*n* = 6/group). The concentration of sodium propionate was selected based on previous studies (Qian et al., 2024). Mice in the SP and control groups were administered sodium propionate (Sigma, USA, P1880) at 200 mg/kg or an equal volume of 0.9% saline, respectively, by daily oral gavage. The treatment was sustained for 21 days, and the body weight of each mouse was recorded daily. At the end of the experiment, all mice were euthanized humanely by cervical dislocation, and then blood, colonic contents and colonic tissues were collected.

### 2.2 Intestinal histology analysis

The intestines preserved in 4% paraformaldehyde were embedded in paraffin. A total of 5 μm sections were cut and dyed with hematoxylin and eosin (H&E). The number of goblet cells in intestinal segments was counted by periodic acid Schiff (PAS).

### 2.3 16 S rRNA gene sequencing

The colonic contents were collected and immediately snap frozen in liquid nitrogen, and then stored at −80 °C. Microbial genomic DNA was extracted using the HiPure Stool DNA Kits fecal DNA extraction kit (Magen, China, D3141). The V3–V4 region of 16S rRNA gene was amplified by PCR with specific primers (341F: 5′-CCTACGGGNGGCWGCAG-3′ and 806R: 5′-GGACTACHVGGGTATCTAAT-3′). Purified products were sequenced on the Illumina Novaseq 6000 PE250 platform (Illumina, USA). Raw data was primarily filtered by Trimmomatic (version 0.33) (Bolger et al., 2014). Identification and removal of primer sequences was process by Cutadapt (version 1.9.1). Clean reads obtained from previous steps were assembled by USEARCH (version 10) under the following settings: minimum overlap length of 10 bp; minimum overlap similarity of 90%; maximum accepted mismatches of 5 bp (default) (Edgar, 2013) and followed by chimera removal using UCHIME (version 8.1) (Edgar et al., 2011). DADA2 method in QIIME2 (versoin 2020.06) (Callahan et al., 2016) was applied to de-noise sequences, generating ASVs. Conservative threshold for OTU filtration is 0.005%. Species annotation and taxonomic analysis were based on DatabaseSilva (Quast et al., 2013). Alpha-diversity of the microbial community was assessed using Mothur (v1.30) by employing indices including Chao1, Shannon, and Simpson. Bray-Curtis distances were used to quantify community composition differences, followed by visualization with principal coordinate analysis (PCoA) and non-metric multidimensional scaling (NMDS) using QIIME 2. STAMP was used to analyze the differential microbial communities. The correlation network of dominant bacterial genera was visualized with Cytoscape based on Spearman analysis.

### 2.4 Measurement of short-chain fatty acids concentration in the colon

The concentrations were measured using gas chromatography Briefly, take 0.1 g sample in a 1.5 mL centrifuge tube, add 500 μL of water and 100 mg of glass beads, homogenize for 1 min, centrifuge at 4 °C and 12,000 rpm for 10 min, take 200 μL of the supernatant, add 100 μL of 15% phosphoric acid, then add 20 μL of 375 μg/mL 4-methylvaleric acid solution and 280 μL of ethyl ether, homogenize for 1 min, centrifuge at 4 °C and 12,000 rpm for 10 min, and take the supernatant for instrumental analysis. The chromatographic column used is an Agilent HP-INNOWAX capillary column (30 m × 0.25 mm ID × 0.25 μm); the injection port temperature is 250 °C. The carrier gas is helium, with a carrier gas flow rate of 1.0 mL/min.

### 2.5 Immunohistochemistry

The tissue sections were deparaffinized and antigen retrieval by heating in 10 mM citrate buffer (pH 6.0) using a water bath at 98 °C for 20 min, followed by natural cooling to room temperature. Subsequently, endogenous peroxidase activity was quenched by incubation in 3% H_2_O_2_ for 25 min. and blocked in PBS with 3% BSA at room temperature for 30 min. The sections were incubated with primary antibody including IL-6 (1:500, Servicebio, China, GB11117), IL-10 (1:500, Servicebio, China, GB11108), and occludin (1:500, Servicebio, China, GB11149) at 4 °C overnight. After washing, the sections were incubated with HRP conjugated goat anti-rabbit IgG (H + L) (1:1000, Servicebio, China, GB23303). The DAB (KIT-9901, Elivision TM plus Polyer HRP IHC Kit, Fuzhou, China) solution was added to the sections for immunohistochemical staining. Images were acquired under an OLYMPUS BX53 microscope and analyzed with ImageJ software. Quantitative analysis was performed using ImageJ software by measuring the integrated optical density (IOD) of positive staining per section.

### 2.6 Hematology

Anticoagulated blood sample was collected from the retro-orbital sinus post-experiment. Routine blood testing was carried out using a fully automated blood analyzer (Mindray Medical, China). White blood cells (WBC), neutrophils (Neu), lymphocytes (Lym), monocytes (Mon), eosinophils (Eos), basophils (Bas), % neutrophil (Neu%), % lymphocyte (Lym%), % monocyte (Mon%), % eosinophil (Eos%), % basophil (Bas%), red blood cells (RBC), hemoglobin (HGB), hematocrit (HCT), mean corpuscular volume (MCV), mean corpuscular hemoglobin (MCH), mean corpuscular hemoglobin concentration (MCHC), red cell distribution width-coefficient of variation (RDW-CV), red cell distribution width-standard deviation (RDW-SD), platelets (PLT), mean platelet volume (MPV), platelet distribution width (PDW), plateletcrit (PCT) were measured.

### 2.7 Statistical analysis

The data analyses were performed with SPSS 22.0 software (IBM Corp.). Inter-group differences for continuous variables (e.g., body weight, hematology, histology) were compared using the Independent-samples *t*-test or the Mann-Whitney U test, based on normality and variance homogeneity. To account for multiple testing, the significance level for pre-specified key outcomes was adjusted using the Bonferroni method, while the False Discovery Rate (FDR) was controlled for microbiome data using the Benjamini-Hochberg procedure. Effect sizes (Cohen’s d or r) were also reported to complement *P*-values. Statistical significance was defined as a Bonferroni-adjusted *P*-value or an FDR *q*-value of <0.05.

## 3 Results

### 3.1 Effects of sodium propionate on body weight, and histopathology in mice

**FIGURE 1:**
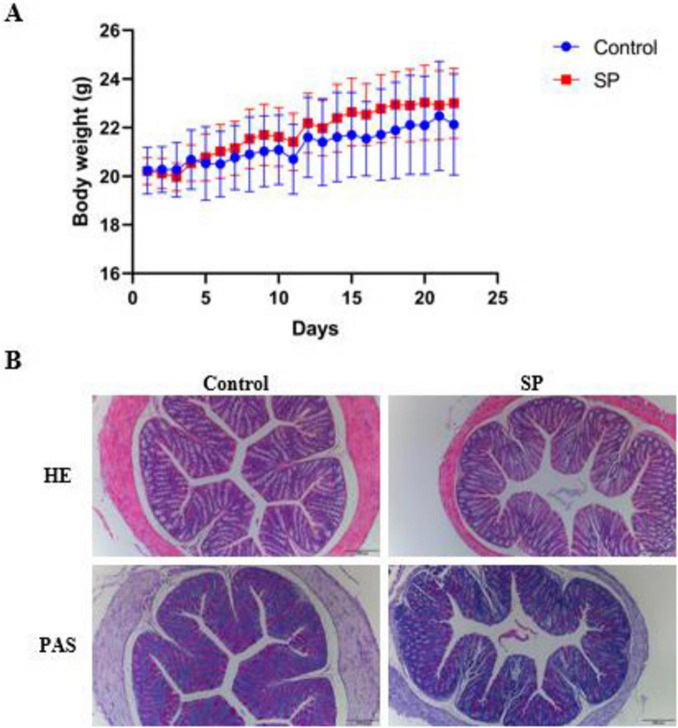
Effects of sodium propionate on body weight, and histopathology in mice. **(A)** Body weight changes; **(B)** Representative hematoxylin and eosin (H&E) and periodic acid Schiff (PAS) images of colon tissue.

The body weight of mice in the SP group was higher than that of the Control group starting from day 5, but no significant difference was found between the two groups during the experimental period (*P* > 0.05) (Figure 1A). The results of HE and PAS staining revealed that colonic tissues in both the Control and SP groups displayed intact morphology and abundant goblet cells (Figure 1B).

### 3.2 Sodium propionate alters blood parameters in mice

The effects of SP on blood parameters are summarized in Table 1. Compared with the control group, the sodium propionate group showed no significant differences in all hematological parameters (including RDW-CV, RDW-SD, PLT, and PCT; *P* > 0.05). The results indicate that, under the experimental conditions of this study, sodium propionate had no significant effect on the hematological parameters of normal mice.

**TABLE 1:** Blood routine results of mice in two groups.

Index	Control	DSS
WBC (×10^9^/L)	8.05 ± 1.81	6.63 ± 2.27
Neu (×10^9^/L)	0.76 ± 0.27	0.79 ± 0.44
Lym (×10^9^/L)	6.91 ± 1.45	5.55 ± 1.76
Mon (×10^9^/L)	0.29 ± 0.12	0.24 ± 0.1
Eos (×10^9^/L)	0.07 ± 0.04	0.04 ± 0.02
Bas (×10^9^/L)	0.01 ± 0.01	0.01 ± 0.01
Neu% (%)	9.2 ± 2.04	11.28 ± 2.59
Lym% (%)	86.23 ± 3.22	84.4 ± 2.91
Mon% (%)	3.52 ± 1.04	3.58 ± 1.43
Eos% (%)	0.92 ± 0.41	0.57 ± 0.2
Bas% (%)	0.13 ± 0.05	0.17 ± 0.12
RBC (×10^12^/L)	10.43 ± 0.2	10.35 ± 0.44
HGB (g/L)	164.67 ± 5.09	161.67 ± 7.09
HCT (%)	48.15 ± 0.89	47.47 ± 2.38
MCV (fL)	46.15 ± 0.18	45.87 ± 0.34
MCH (pg)	15.75 ± 0.26	15.63 ± 0.18
MCHC (g/L)	341.67 ± 6.02	340.83 ± 3.76
RDW-CV (%)	14.63 ± 0.33	13.93 ± 0.48
RDW-SD (fL)	28.33 ± 0.84	26.7 ± 1.01
PLT (×10^9^/L)	184.5 ± 37.85	124.5 ± 44.42
MPV (fL)	5.42 ± 0.12	5.33 ± 0.14
PDW	15.5 ± 0.22	15.87 ± 0.45
PCT (%)	0.13 ± 0.03	0.13 ± 0.16

### 3.3 Effects of sodium propionate on the expression of IL-6, IL-10, and occludin in mice

**FIGURE 2:**
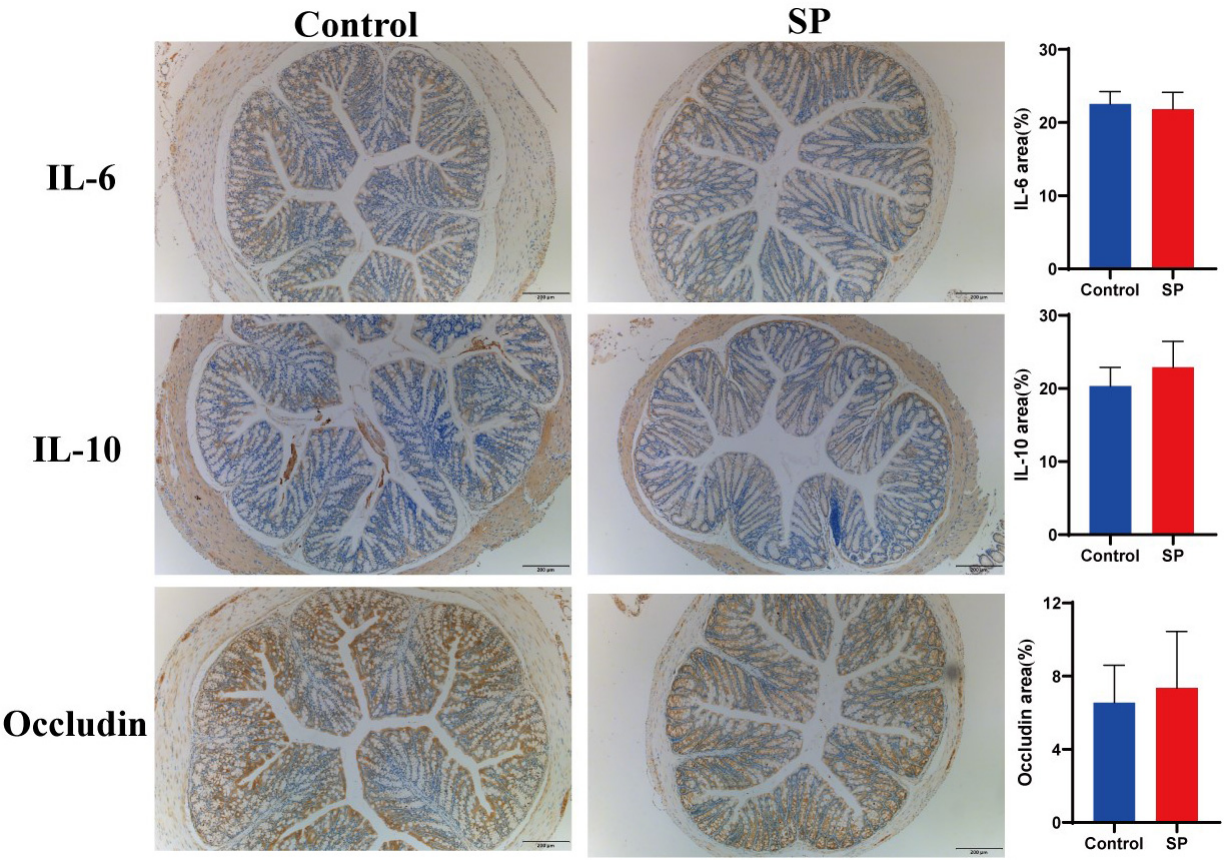
Effects of sodium propionate on IL-6, IL-10, and occludin proteins expression of colon tissue.

Immunohistochemistry (IHC) staining results showed that IL-6 expression was decreased in the SP group compared to the control, whereas IL-10 and occludin expression were increased (*P* > 0.05). This altered expression profile was consistent with the preservation of intestinal structural integrity in the SP group (Figure 2).

### 3.4 Effects of sodium propionate on the composition and diversity of gut microbiota in mice

**FIGURE 3:**
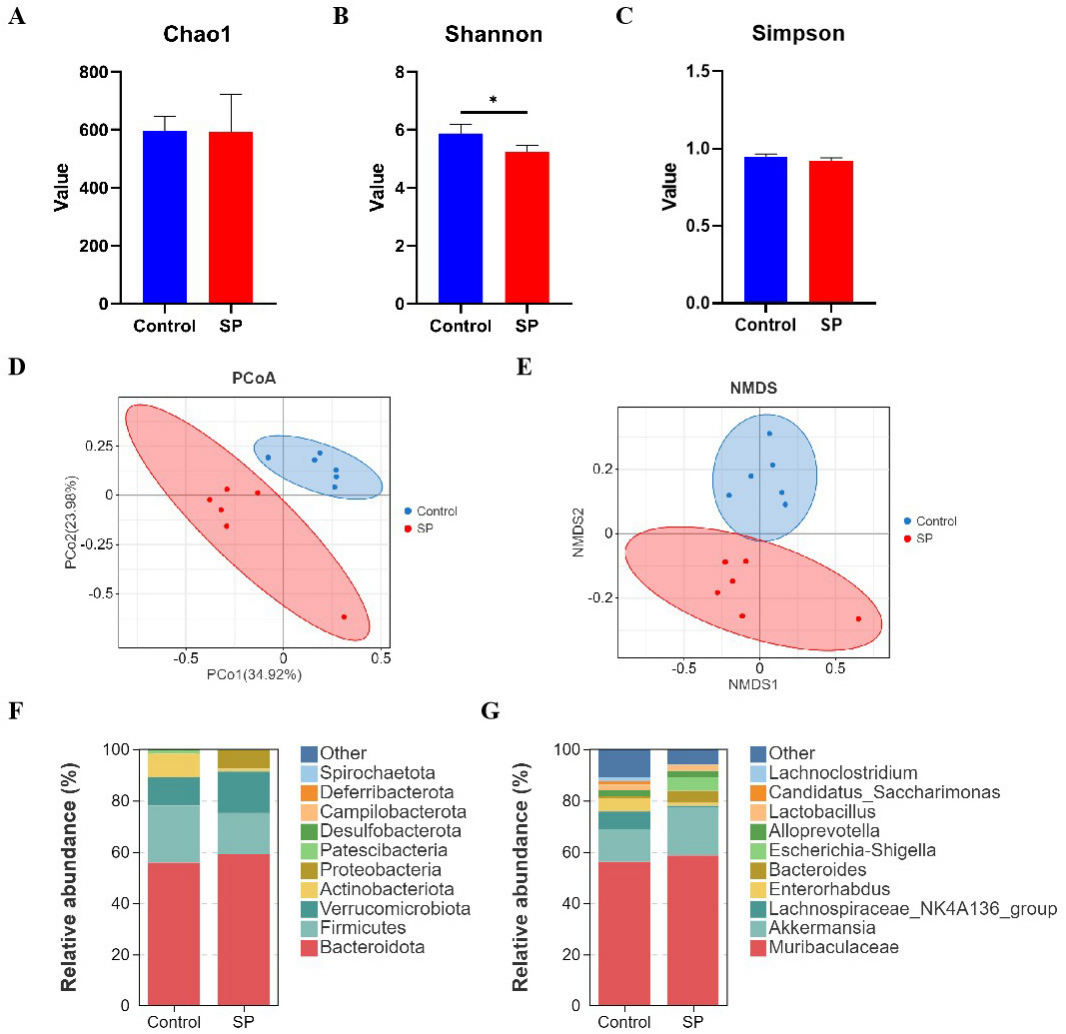
Diversity and composition of gut microbiota in two groups of mice. **(A–C)** Analysis of α-diversity in the gut microbiota of mice under different treatments; **(D,E)** Analysis of β-diversity in the gut microbiota of mice under different treatments; **(F,G)** Composition of gut microbiota at phylum and genus levels in mice under different treatments. *Indicates a statistically significant difference, with *P < 0.05.

No significant differences were observed in the Chao1, Shannon, and Simpson indexes between the Control group and the SP group. However, the Shannon and Simpson indexes of the Control group was significantly higher than that of the SP group (*P* < 0.05) (Figure 3A–C). PCoA and NMDS based on Bray-Curtis distance were performed to analyze the β-diversity of gut microbial composition between the two groups (Figures 3D, E). The compositions of the gut microbiota between Control group and SP group were different.

At the phylum level, the top 10 microorganisms in relative abundance in both the Control group and SP group are Bacteroidota, Firmicutes, Verrucomicrobiota, Actinobacteriota, Proteobacteria, Patescibacteria, Desulfobacterota, Campilobacterota, Deferribacterota, and Spirochaetota. Among these, Bacteroidota, Firmicutes, and Verrucomicrobiota are the dominant phyla in both the Control and SP groups. The relative abundance of Firmicutes was higher in the Control group than in the SP group. The relative abundances of Bacteroidota and Verrucomicrobiota were higher in the SP group than in the Control group (Figure 3F). At the genus level, the top 10 microorganisms in relative abundance in both the Control and SP groups were *Muribaculaceae*, *Akkermansia*, *Lachnospiraceae_NK4A136_group*, *Enterorhabdus*, *Bacteroides*, *Escherichia-Shigella*, *Alloprevotella*, *Lactobacillus*, *Candidatus_Saccharimonas*, and *Lachnoclostridium*. The relative abundance of *Lachnospiraceae_NK4A136_group*, *Enterorhabdus*, *Candidatus_Saccharimonas*, and *Lachnoclostridium* was higher in the Control group than in the SP group. The relative abundances of *Muribaculaceae*, *Akkermansia*, and *Bacteroides* were higher in the SP group than in the Control group (Figure 3G).

**FIGURE 4:**
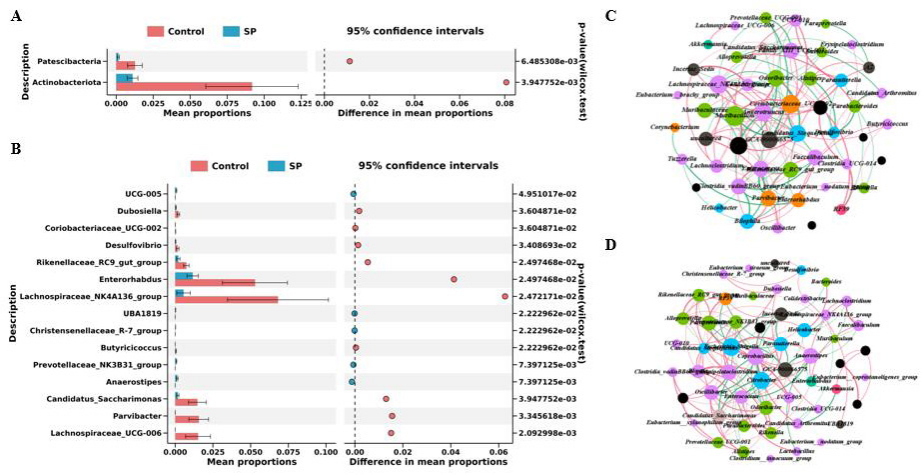
Microbiome differential analysis. **(A)** Significantly different microbial taxa at the phylum level in mice across treatments; **(B)** Significantly different microbial taxa at the genus level in mice across treatments; **(C)** Analysis of the intestinal microbiota interaction network in the Control group of mice; **(D)** Analysis of the intestinal microbiota interaction network in the SP group of mice.

This study further analyzed the statistically significant differences in microbial composition between the Control and SP groups. At the phylum level, the relative abundances of Actinobacteriota and Patescibacteria were significantly lower in the SP group compared to the Control group (*P* < 0.05, *q*-value < 0.05) (Figure 4A). At the genus level, significant differences (*P* < 0.05, *q*-value < 0.05) were observed in the relative abundances of 15 bacterial genera between the Control and SP groups. Among these, the relative abundances of *Lachnospiraceae_UCG-006*, *Parvibacter*, *Candidatus_Saccharimonas*, *Lachnospiraceae_NK4A136_group*, *Enterorhabdus*, *Rikenellaceae_RC9_gut_group*, *Desulfovibrio*, *Coriobacteriaceae_UCG-002*, and *Dubosiella* were significantly higher in the Control group than in the SP group (*P* < 0.05, *q*-value < 0.05). Additionally, the relative abundances of *Anaerostipes*, *Prevotellaceae_NK3B31_group*, *Christensenellaceae_R-7_group*, *UBA1819*, and *UCG-005* were significantly lower in the Control group than in the SP group (*P* < 0.05). The genus *Butyricicoccus* showed a trend toward lower abundance in the Control group but did not reach statistical significance after FDR correction (*P* < 0.05, *q*-value = 0.055) (Figure 4B).

We constructed intestinal microbial interaction networks for the two mouse groups. Analysis of the microbial interaction networks revealed that the network in the SP group exhibited greater complexity. Core microbes (genera interacting with other microbes) differed between the two groups. *Lachnospiraceae_NK4A136_group*, *Anaerotruncus*, and *Coriobacteriaceae_UCG-002* were unique hub microbes in the Control group (Figure 4C), whereas *Citrobacter*, *Oscillibacter*, and *Erysipelatoclostridium* were unique hub microbes in the SP group (Figure 4D).

### 3.5 Analysis of short-chain fatty acids in mice

**FIGURE 5:**
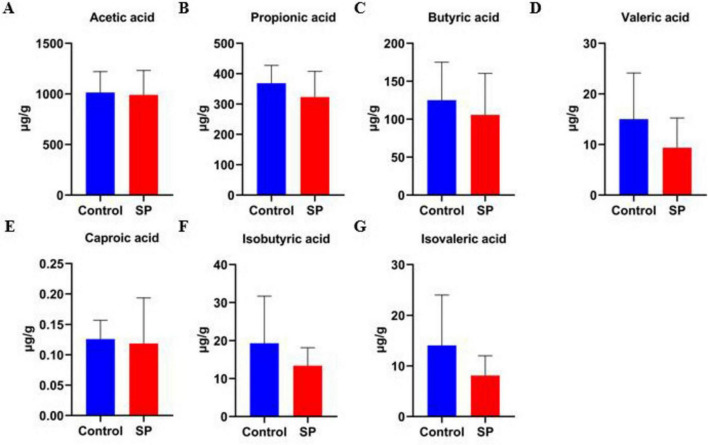
Impact of sodium propionate gavage on colonic short-chain fatty acids (SCFA) levels in mice. **(A)** Acetic acid; **(B)** Propionic acid; **(C)** Butyric acid; **(D)** Valeric acid; **(E)** Caproic acid; **(F)** Isobutyric acid; **(G)** Isovaleric acid.

We quantified the concentrations of seven short-chain fatty acids (SCFAs) in mouse colon contents. Although the Control group exhibited a trend of higher concentrations across all SCFAs, including acetic acid, propionic acid, butyric acid, valeric acid, caproic acid, isobutyric acid, and isovaleric acid, compared to the SP group, these differences did not reach statistical significance (*P* > 0.05) (Figure 5).

## 4 Discussion

Short-chain fatty acids (SCFAs) are increasingly recognized for their regulatory roles in metabolism, immunity, and gut health. While previous studies have focused on propionate’s therapeutic effects in disease models, its impact on healthy organisms remains further exploration (Bartolomaeus et al., 2019; Pham et al., 2021; Yan et al., 2022; Chandra et al., 2025). This study systematically assessed the physiological effects of sodium propionate supplementation in healthy mice, revealing alterations in gut microbiota composition and hematological parameters without intestinal histological changes. Our findings indicate that SP administration did not significantly affect body weight or intestinal morphology, suggesting that propionate does not induce adverse effects in healthy mice.

While no statistically significant differences in hematological parameters were observed in the present study, some observed numerical trends merit cautious interpretation in the context of existing literature. Elevated WBC is a biomarker of ulcerative colitis (Cioffi et al., 2015). SP reduced WBC in mice, suggesting that oral sodium propionate did not induce systemic inflammation. Similarly, numerical decreases were noted in RDW-CV, RDW-SD, PLT, and PCT, suggesting altered erythropoiesis or platelet production. Lower RDW may reflect improved iron utilization (Hanawa et al., 2025). Additionally, increased RDW has been recognized as a novel inflammatory biomarker for various diseases (Pena-Duran et al., 2025). This may be related to the role of hepcidin. Hepcidin regulates iron homeostasis by reducing serum iron levels, ultimately leading to impaired erythropoiesis and increased RDW (Huang and Kuo, 2017). PLT and PCT platelet indicators reflect platelet biochemical and functional changes, playing key roles in immune and inflammatory responses (Li and Liu, 2024). Previous studies have found that PLT and PCT are significantly elevated in IBD patients than healthy individuals (Xu et al., 2024). Therefore, the observed non-significant trends of reduction in these parameters might hint at a potential, albeit unconfirmed, modulatory effect of sodium propionate on inflammatory pathways. Future studies with larger sample sizes are required to substantiate whether these trends represent a genuine biological effect.

Pro-inflammatory cytokines are closely associated with the pathogenesis of colitis (Ahmadi et al., 2025), and their levels positively correlate with disease severity (Cerf-Bensussan and Gaboriau-Routhiau, 2010). In this study, sodium propionate treatment reduced the protein expression of the pro-inflammatory cytokine IL-6 while increasing that of the anti-inflammatory cytokine IL-10 in colonic tissues. These results are consistent with prior reports on the anti-inflammatory properties of sodium propionate (Tong et al., 2016). This suggests that sodium propionate may exert anti-inflammatory effect by modulating leukocyte/lymphocyte/macrophage functions. Occludin is a critical component of the intestinal barrier. Overexpression of occludin has been shown to enhance epithelial barrier function *in vitro* (McCarthy et al., 1996). Although sodium propionate did not significantly increase occludin levels in healthy mice in this study, previous studies have reported that it can restore occludin levels in mice with colitis (Tong et al., 2016). This may reflect its predominant protective effects during inflammatory states rather than basal conditions. Future studies should further investigate the effects of sodium propionate on gut microbiota-immune interactions to elucidate its potential therapeutic value in IBD treatment.

At the phylum level, Firmicutes and Bacteroidota were the dominant bacterial phyla in the mouse gut, which is consistent with previous findings (Liu et al., 2023). These two phyla represent core microbial components of the mammalian gastrointestinal tract (Chen et al., 2022). At the genus level, the most relatively abundant bacterial genus is *Muribaculaceae*. In dyslipidemic mice, the relative abundance of *Muribaculaceae* decreases, leading to reduced intestinal hyodeoxycholic acid (HDCA) levels, increased bile acid synthesis, and abnormal lipid metabolism (Xu et al., 2023). *Muribaculaceae* can improve the intestinal barrier and modulating immune responses. It is regarded as a “next-generation probiotic,” and its increased abundance is negatively correlated with inflammatory bowel disease, type 2 diabetes, and other conditions (Zhu et al., 2024). The SP group exhibited reduced microbial diversity. Sodium propionate may not exert its therapeutic effects by altering the diversity or richness of the colonic microbiota in colitis mice (Liu et al., 2023). Another study demonstrated that sodium butyrate had no significant effects on the richness, diversity, or evenness of the animal gut microbiota (Luo et al., 2023).

Relative abundances of *Akkermansia*, *Anaerostipes*, *Prevotellaceae_NK3B31_group*, *Christensenellaceae_R-7_group*, *UBA1819*, and *UCG-005* were increased in the SP group. The genus *Akkermansia* is a prevalent gut microbe whose primary function involves degrading host mucins into various metabolites, thereby maintaining intestinal barrier integrity and modulating immune responses (Ding Y. et al., 2025). *Anaerostipes* can metabolize inositol-containing substrates, thereby reducing the risk of metabolic disorders in the host and potentially exerting beneficial effects on host health (Bui et al., 2021). Oral administration of sodium propionate significantly increased the relative abundance of the *Prevotellaceae_NK3B31 group*, suggesting potential alterations in host metabolism, including amino acid, carbohydrate, lipid, and nucleotide metabolism (Jiang et al., 2020). Additionally, *Christensenellaceae R-7 group* suppresses inflammatory responses via regulation of the MyD88 pathway (Gao et al., 2023). *UBA1819* and *UCG-005* may serve as potential probiotics to reduce or prevent diarrhea in calves (Chen et al., 2022). This is also consistent with the absence of observed diarrhea in mice during our experimental period. These changes suggest that propionate may selectively enrich beneficial microbes (Yan et al., 2022).

As individual metabolic processes become more active and growth rates increase, the ecological network will grow in complexity (Stark et al., 2025). Microbial interaction networks in the SP group were more complex, with unique hub genera indicating potential functional shifts in microbial metabolism. This reflects the mouse’s adaptive response to sodium propionate supplementation. Sodium propionate intervention reshaped the gut microbiota of mice, enriching beneficial genera such as *Akkermansia* and *Anaerostipes*. These changes coincided with intestinal anti-inflammatory and barrier-strengthening effects, accompanied by improvements in systemic inflammatory markers, suggesting that local intestinal amelioration can systemically promote immune homeostasis (Sen, 2025). We speculate that sodium propionate plays a central role in mediating these effects by activating G protein-coupled receptors (GPR41/43) and inhibiting the NF-κB pathway (Hsu et al., 2025; Wang et al., 2025). This mechanism not only directly suppresses local intestinal inflammation but may also reduce the leakage of pro-inflammatory factors into the systemic circulation by reinforcing barrier integrity, thereby systemically alleviating the inflammatory state (Ding W. et al., 2025).

Sodium propionate supplementation did not significantly alter colonic SCFA concentrations. A key reason for this may be the efficient pre-colonic absorption and first-pass metabolism. Orally administered SCFAs are rapidly absorbed in the upper gastrointestinal tract, primarily in the small intestine, via specific transporters such as MCT1 and SMCT1 (Ziegler et al., 2016). The absorbed propionate then enters the portal vein circulation and is largely extracted and metabolized by the liver for processes such as gluconeogenesis before reaching the systemic circulation (den Besten et al., 2013; Kennedy et al., 2020). This process significantly reduces the total amount of the administered dose that reaches the colonic lumen. Secondly, the exogenous propionate that does reach the colon may act as a signal, inhibiting the fermentative activity of key SCFA-producing bacteria such as Bacteroides and Veillonella (Nireeksha et al., 2025). Finally, the dynamic homeostasis of the colonic ecosystem must be considered. The colonic SCFA pool is the result of continuous microbial production, host absorption, and utilization by other microbes. The colon can rapidly upregulate SCFA absorption via the same transporters (MCT1/SMCT1), thereby effectively maintaining a stable luminal environment (Sivaprakasam et al., 2017). In summary, the lack of change in colonic SCFA levels does not indicate that the intervention was ineffective but rather reflects the complex physiology of host and microbial regulation. The biological effects of oral sodium propionate are more likely mediated through systemic pathways, such as the activation of portal or hepatic nutrient-sensing mechanisms and GPR receptor signaling, rather than through significant alterations in the local colonic SCFA pool (De Vadder et al., 2014).

This study had several limitations. First, the duration of SP treatment (21 days) might have been insufficient to induce pronounced metabolic or immunological changes. Future studies could explore longer intervention periods, and multi-omics approaches are needed to elucidate propionate’s mechanisms. Furthermore, investigation in germ-free mouse models could help differentiate between propionate’s direct host effects and microbiota-dependent mechanisms.

## 5 Conclusion

In summary, this study demonstrates that propionate supplementation in healthy mice modulates gut microbiota composition and hematological parameters. Propionate supplementation reduced IL-6 expression while increasing IL-10 and occludin expression. These findings provide a foundation for future research on propionate’s physiological roles and its potential applications in preventive health strategies.

## Data Availability

The data presented in the study are deposited in the NCBI repository, accession number PRJNA1380418.
